# Amplitude and phase control in SIW structures by tuning multilayered graphene

**DOI:** 10.1038/s41598-025-26818-z

**Published:** 2025-11-04

**Authors:** Shakir Ullah, Muhammad Yasir

**Affiliations:** Department of Computing Sciences, University of Oldenburg, Oldenburg, Germany

**Keywords:** Graphene, Graphene microwave, Gigahertz (GHz), Dynamic range, Tunable graphene, Phase variation, Quadrature amplitude modulation (QAM), Graphene half mode substrate integrated waveguide (GHMSIW), Engineering, Materials science, Nanoscience and technology, Optics and photonics, Physics

## Abstract

Graphene is a unique 2D material that provides exceptional mechanical properties, ultra-high carrier mobility, and dynamically adjustable surface conductivity, making it an excellent choice for microwave devices. However, the dynamic manipulation of amplitude and phase in substrate integrated waveguides based on multilayered graphene has not been experimentally proven. In this paper, we propose a novel multilayered graphene based half mode SIW attenuator and phase shifter for the manipulation of amplitude and phase at microwave frequencies. The amplitude and phase are controlled by applying a bias voltage to graphene. The applied voltage is increased from 0 to 6.2 V, and the sheet resistance (Ω/□) of graphene can be tuned between 1302 and 60 Ω/□, depending on the gap size for deposition of graphene. The simulated and measured results of the proposed attenuator and phase shifter demonstrate the manipulation of microwave signals. The dynamic range achieved for amplitude is 13dB, and that of phase is 45 degrees. The proposed technique proves that the amplitude and phase of microwave signals can be tuned in SIW based devices without any localized surface mounted devices (SMD) or complex realization techniques.

## Introduction

Graphene is a 2-D material arranged in a hexagonal shape like a honeycomb lattice. Every carbon atom in graphene is attached to other three neighboring carbon atoms with covalent bonds. Each carbon atom has a free electron that is not involved in covalent bond. The free electrons contribute to an increased surface conductivity of graphene. This makes graphene an exceptional material for future electronics^1^. In recent years, an increasing number of researchers have made significant progress in microwave applications, benefiting from graphene’s unique characteristics. These include high electron mobility, light weight, mechanical strength, impermeability, planarization, and flexibility compared to conventional semiconducting materials^2^and^3^. The most interesting property of graphene at microwave frequencies is its tunable electrical conductivity. The electrical conductivity of graphene can be varied over a large range by applying a DC voltage. This is the reason why graphene has been used in a number of components e.g., phase shifter, antenna, power dividers, attenuators, and metasurface^4–8^.

SIW technology has the same characteristics as dielectric filled waveguides^9^. Thus, SIW possesses the behavior of both microstrip line and waveguide technologies. Besides SIW has the capability of handling high power, and unlike microstrip lines, SIW confines the EM field within the substrate, minimizing leakage and interference. This is crucial for mobile, aerospace, terrestrial 5G communications, satellite systems, automotive radars, and IOT connectivity, where space and weight are desired. SIW technology is used in sensor development for material characterization and biomedical applications^10–12^. In light of the above applications, this work offers a scalable, low-loss, and integration-friendly path to advanced RF components.

Attenuator is an important microwave component used in applications that demand limiting the power of the transmitted signal. To reduce return loss and facilitate convenient integration with planar integrated circuits, SIW-based attenuator is an optimal choice. The attenuators reported^13^ and^14^ are based on microstrip and GCPW technologies with multilayered graphene. While the microstrip technology has limitations related to power handling, the SIW attenuators reported so far are based on high technological complexity. In the SIW based attenuators reported in^15^ and^16^, the tuning element is based on graphene sandwich structure (GSS) with monolayer graphene.

Phase shifters are key components for modulation and demodulation in radio frequency circuits. Conventionally, PIN diodes are used in phase shifting applications. The use of PIN diodes in phase shifters introduces unwanted design complexities. Additionally, PIN diodes have high insertion loss and are difficult to integrate in conventional fabrication processes^17,18^. More recently, liquid metals vias have been used as switching elements in SIW phase shifters^19^ and ^20^. Unlike electronically tunable elements such as graphene, liquid metal vias function through the physical injection or removal of metal, allowing for discrete instead of continuous phase tuning. Nevertheless, the fabrication of graphene-based diodes is more complicated as compared to the fabrication of graphene based microwave components e.g., graphene based attenuators and phase shifters.

To simplify the fabrication process of attenuators and phase shifters, this paper introduces a novel solution using multilayered graphene in SIW technology at microwave frequencies for the first time. Multilayered graphene is easy to deposit on the designated location and provides increased values of electrical conductivity. This results in a reduced cost of deposition and ease of fabrication as compared to monolayer graphene GSS based components. The proposed attenuator and phase shifter are appropriate for integration in substrate integrated waveguide components utilizing multilayered graphene as a tunable component.

This paper is organized as follows: The design principles and operation of the attenuator and phase shifter are explained in Sect. Design strategy and operation of Ghmsiw attenuator and phase shifter. The fabrication and measurement processes of the attenuator and phase shifter are presented in Sect. Fabrication and measurement setup of the attenuator and phase shifter. Section Results and discussions of the attenuator and phase shifter contains the explanation of the simulation and measurement. The last section contains the discussion and future directions.

## Design strategy and operation of Ghmsiw attenuator and phase shifter

**Fig. 1:**
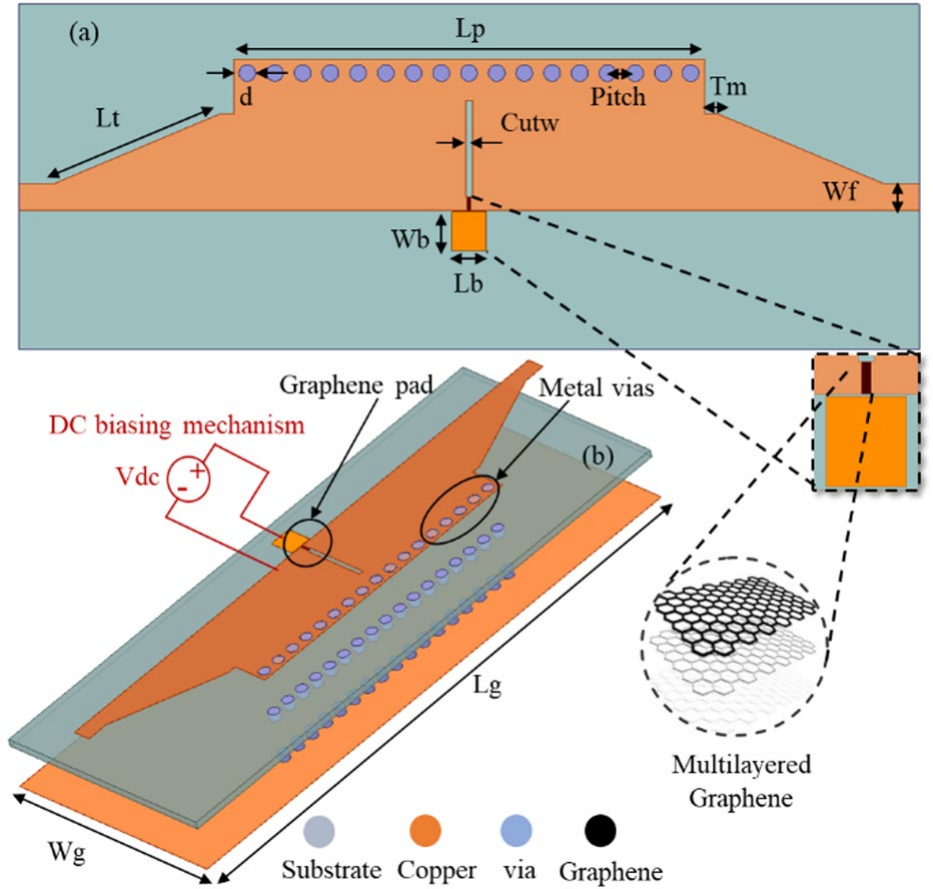
The schematic of the proposed GHMSIW attenuator (**a**) Top view (**b**) 3D view with a DC biasing mechanism for measurements.

The topology of the GHMSIW attenuator is shown in Fig. 1. The attenuator is simulated by the finite element simulation tool, Ansys High Frequency Structure (HFSS) version 2024. The proposed GHMSIW attenuator operates at the wideband of 5.5 GHz to 8.5 GHz. The GHMSIW attenuator comprises of a simple half mode SIW transmission line with a gap of 8 mm×0.5 mm at the center of the line. The gap for graphene deposition is 1 mm×0.3 mm according to the desired aspect ratio. There is a biasing pad close to the gap of the graphene. The graphene pad is appropriately positioned to increase attenuation in the transmission coefficient. The electric field intensity is higher at the open end of the HMSIW line.

The higher resistance of graphene blocks the signal transmission, while the lower resistance allows the maximum transmission. SIW based circuits^21^ are also used to derive dielectric permittivity along with other conventional methods^22^. The various design parameters of SIW can be taken from the sensor as in^21^. The cutoff frequency (f_c10_), effective width (w_eff_), diameter (d) of vias, and the distance between the center of the via (pitch) are calculated by using (1) and (2). Graphene is modeled as a lumped resistive element with assigned resistance values ranging from 138 Ω/□ to 1302 Ω/□ based on the measured graphene impedance characterization values found in literature^13^ and^23^.

1 $$\:{\text{f}}_{\text{c10}}=\frac{1}{2\text{W}\sqrt{\mu\:\epsilon\:}}$$

2 $$\:\text{W}\text{siw}=\text{W}+\frac{{d}^{2}}{0.95\text{P}}$$

Where W, fc10, and P represent the effective width, cutoff frequency, and pitch between the centers of the adjacent vias, respectively. The effective W and patch length Lp determine the lower and upper cutoff frequencies of the SIW. The values of d and P are optimized to minimize the radiation losses. The dimensions of the proposed attenuator in Fig. 1 are L_p_, L_t_, W_f_, d, Pitch, W_b_, L_b_, L_g_, W_g_, T_m_, and cutw: 34 mm, 12 mm, 1.94 mm, 0.6 mm, 2 mm, 2.5 mm, 2.5 mm, 65 mm, 25 mm, 0.5 mm, and 1 mm, respectively.

**Fig. 2:**
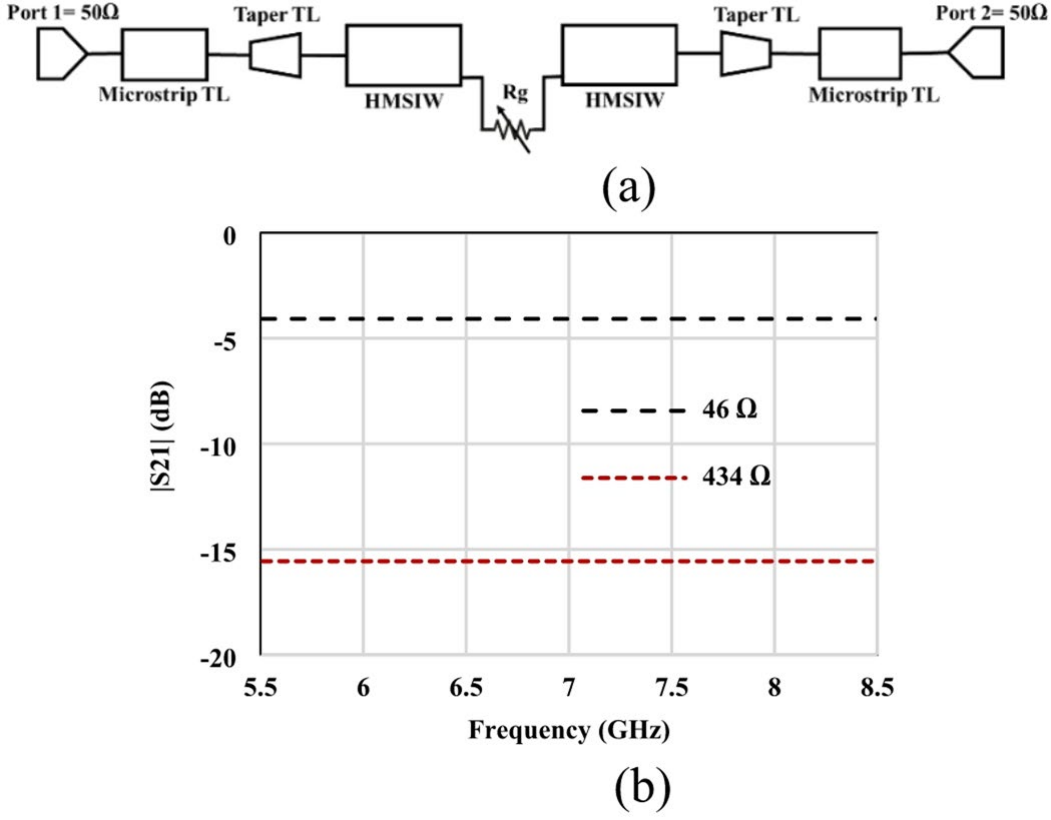
Equivalent circuit model of the GHMSIW attenuator (**a**) Circuit model (**b**) simulated amplitude of the transmission coefficient (S21).

The equivalent circuit model of the proposed HMSIW attenuator is depicted in Fig. 2 (a). The graphene variable resistance Rg is connected in the center between the two HMISW lines. The circuit model consists of two 50 Ω input ports, a microstrip, tapered, and a half-mode SIW transmission line. The resistance of graphene in the equivalent circuit is Rg. The conversion of lumped resistance (Ω) to sheet resistance (Ω/□) values is related to each other through the aspect ratio of the deposited graphene (1 mm x 0.3 mm). The gap size in the direction of current flow is 0.3 mm, so the minimum measured resistance is 46 Ω, and the maximum is 434 Ω (Fig. 2b), with corresponding minimum and maximum sheet resistance values of 138 Ω/□ and 1302 Ω/□, respectively.

**Fig. 3:**
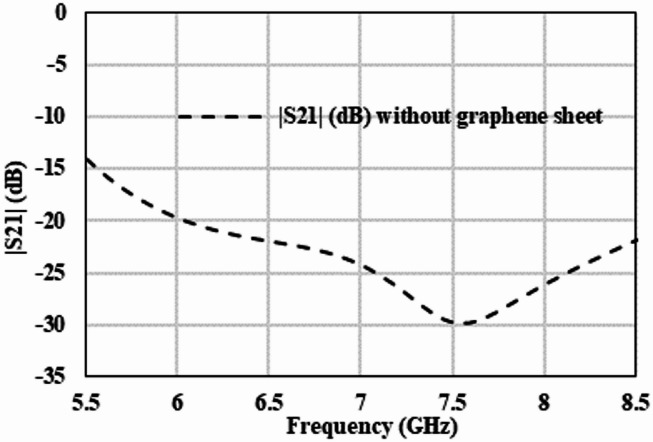
Simulated amplitude of S21 of the attenuator without graphene.

A plot of the S21 is shown in Fig. 3. It is clear that in the absence of graphene, the transmission coefficient is less than − 15dB throughout the entire frequency band. This means that negligible amount of power is transmitted between the two ports, and the majority is attenuated. Thus, it is clear from the empty gap analysis that the gap is large enough to provide negligible transmission. In other words, the parasitic capacitance is negligible according to (3) as in^24^.

3 $$\:\text{C}=\frac{\text{A}.{\upepsilon\:}}{\text{d}}$$

where ε is the relative permittivity of the dielectric, A is the overlapping area, and d is the distance between traces.

**Fig. 4:**
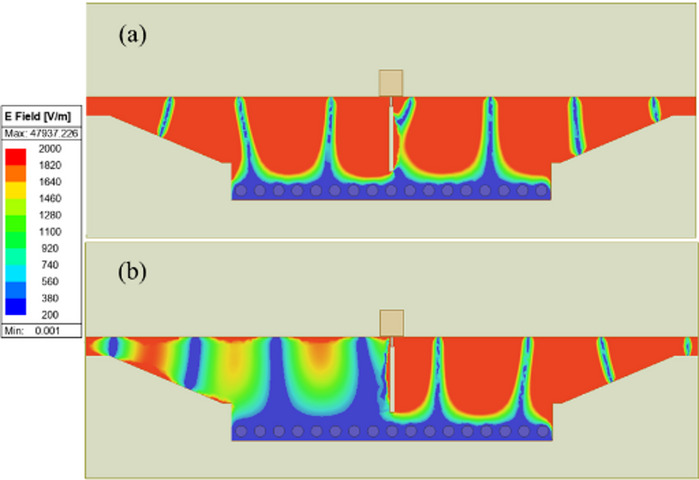
Surface currents distribution of GHMSIW attenuator at 7 GHz with two graphene pads. The graphene surface impedance is (**a**) Rg = 138 Ω/□ and (**b**) Rg 1302 Ω/□.

The electric field distribution of the GHMSIW attenuator is depicted in Fig. 4. The transmission of the signal between the ports can be qualitatively characterized from the electric field distribution. The electric field distribution of GHMSIW shows an increased transmission between port 1 and port 2 for low graphene resistance values (Fig. 4 (a)). For higher resistance of graphene, the signal transmission from port 1 to port 2 is reduced (Fig. 4(b)). This shows that the attenuation can be effectively tuned by changing the resistance values of graphene.

**Fig. 5:**
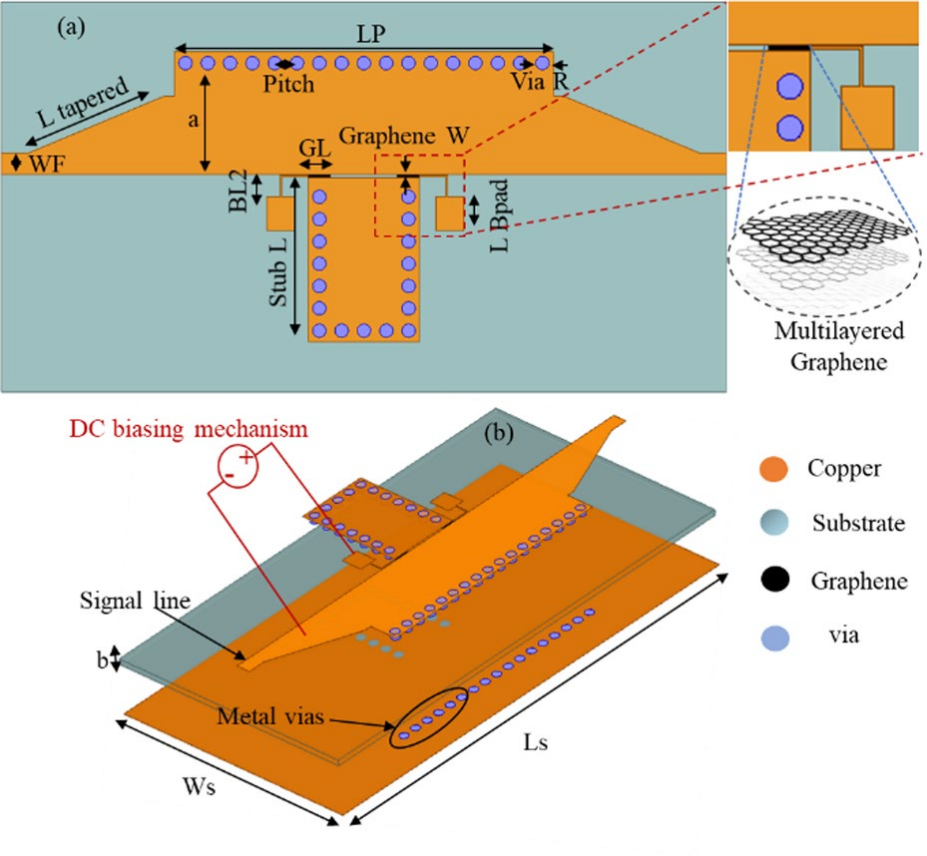
A schematic of the proposed GHMSIW phase shifter (**a**) Top view (**b**) 3D view.

The topology of the GHMSIW phase shifter is shown in Fig. 5. The proposed GHMSIW phase shifter operates in the frequency band of 6.5 to 9.5 GHz with a center frequency of 8 GHz. The GHMSIW phase shifter consists of a half mode SIW transmission line, connected to a full-mode SIW stub through graphene pads. The full mode SIW stub helps in introducing additional phase variation in the transmission coefficient when the resistance of the graphene pads is lowered.

To maximize the flow of the transmitting signal into the stub and the resultant phase variation, the full-mode SIW stub is positioned at the open side of the half-mode SIW transmission line. The cutoff frequencies of a waveguide can be calculated according to the dimensions given in (4) and (5) as in^19^.

4 $$\:{\text{f}}_{\text{c,\:}\text{mn}}=\frac{\text{c}}{2\sqrt{{\epsilon\:}_{r}\:}}\sqrt{({\raisebox{1ex}{$m$}\!\left/\:\!\raisebox{-1ex}{$a$}\right.)}^{2}+({\raisebox{1ex}{$n$}\!\left/\:\!\raisebox{-1ex}{$b$}\right.)}^{2}\:}$$

5 $$\:{\text{f}}_{\text{c,\:m0}}=\frac{\text{m}\text{c}}{2\text{a}\sqrt{{\epsilon\:}_{r}\:}}$$

Where, m, n are the integers, and b represents the height of the SIW between the top and bottom layers, f_c, mn_ are the cutoff frequencies. The effective width (a) determine the lower (6.5 GHz) and upper (9.5 GHz) cutoff frequencies of the TE_10_ and TE_20_ modes of the waveguide. The values of via diameter (d) and pitch (P) are optimized to minimize the radiation losses. The dimensions of the proposed phase shifter in Fig. 5 are L_p_, L_tapered_, W_f_, Via_R_, Pitch, B_L1_, B_L2_, LB_pad_ L_s_, W_s_, G_L_, a, b and L_stub_: 34 mm, 12 mm, 1.94 mm, 0.6 mm, 2 mm, 2.5 mm, 94 mm, 3 mm, 65 mm, 30 mm, 10 mm, 0.813 mm, 0.3 mm, 10 mm, respectively.

**Fig. 6:**
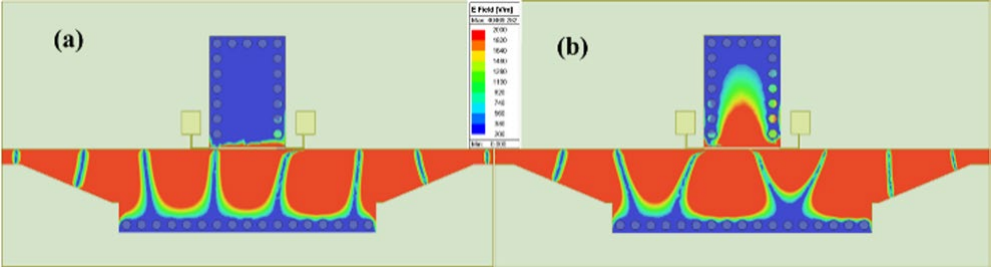
E-filed distribution of GHMSIW phase shifter at 8 GHz with two graphene pads. The graphene surface impedance is (**a**) Rg = 600 Ω/□ and (**b**) Rg = 60 Ω/□.

**Fig. 7:**
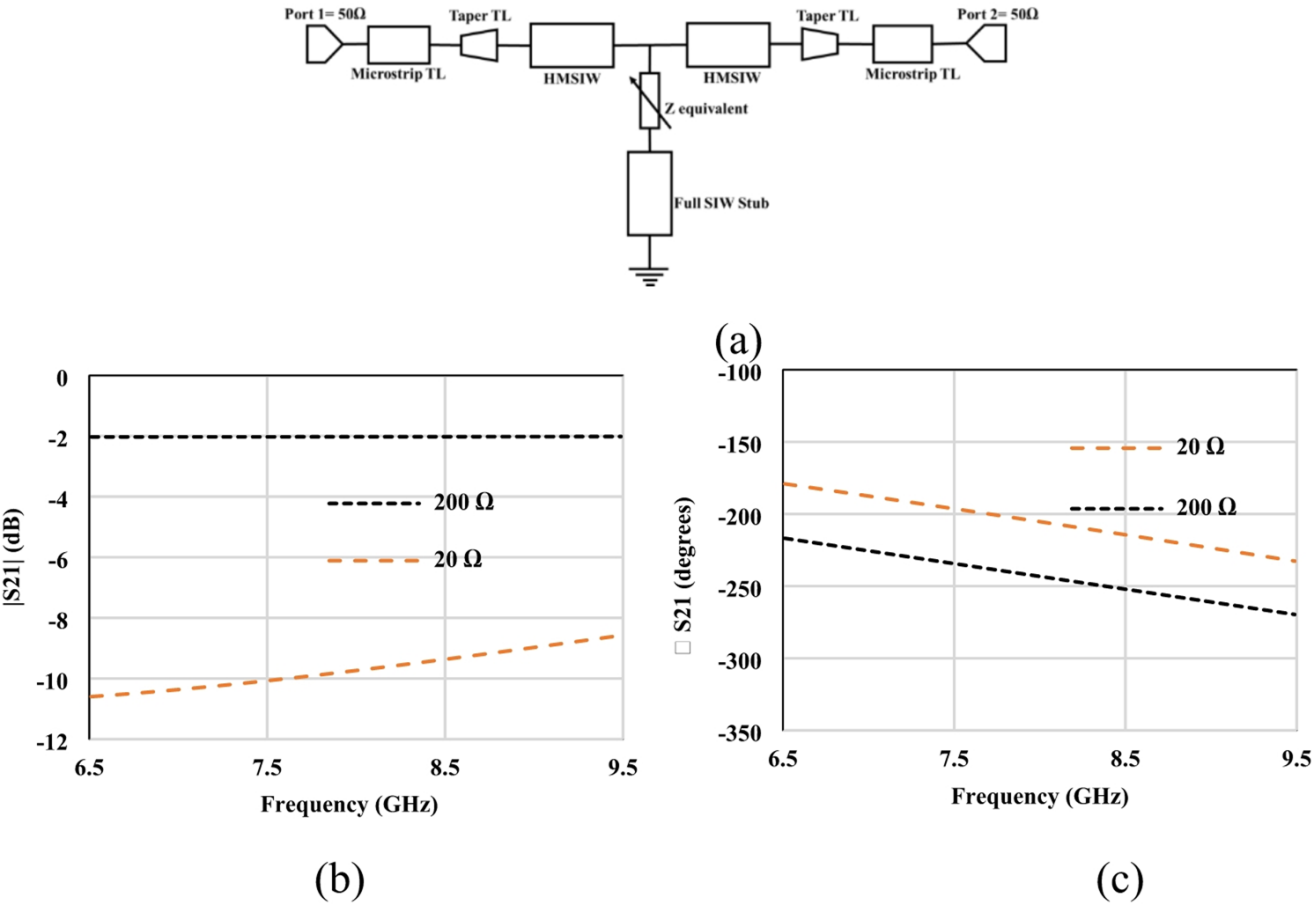
Circuit model analysis of the GHMSIW phase shifter (**a**) Equivalent circuit model (**b**) S_21_ amplitude (**c**) S_21_ phase.

Figure 6 shows the E-field distribution of the proposed phase shifter for different graphene resistance values. For the higher graphene sheet resistance value (Rg = 600 Ω), the GHMSIW phase shifter shows an unperturbed transmission between port 1 and port 2 (see Fig. 6 (a)). The higher resistance of graphene makes this phase shifter a simple half-mode SIW-based transmission line. In the case of lower resistance of the graphene sheet (Rg = 60 Ω), there is an increased phase shift in the transmission of the signal. It is obvious from the E-field distribution that the path for the signal changes and the electrical length of the transmission line increases, causing a delay in the transmission of the signal. For lowered graphene sheet resistance values, the signal passes through the SIW stub, as shown in Fig. 6 (b).

The equivalent circuit model of the proposed HMSIW phase shifter is depicted in Fig. 7 (a). The circuit model consists of two 50Ω input ports, a microstrip, tapered, half-mode SIW transmission line and a stub. The equivalent resistance of the two graphene pads is Req = R1||R2. The simulated amplitude and phase of S21 of the equivalent circuit, which includes 200Ω and 20 Ω graphene resistance, is taken from the sheet resistance adjusted according to the aspect ratio^23^, is shown in Fig. 7 (b) and (c). The lumped resistance (Ω) value is measured first and then converted to sheet resistance (Ω/□) by the aspect ratio of the deposited graphene (1 mm x 0.3 mm). The gap size in the direction of current flow is 0.3 mm, so the measured resistance values 200Ω and 20Ω (Fig. 7b and c) correspond to sheet resistance 600Ω∕□ and 60Ω∕□, respectively.

## Fabrication and measurement setup of the attenuator and phase shifter

The measurements of a multilayered GHMSIW attenuator and phase shifter are performed in the upper C (5.5 GHz to 8 GHz) and lower X (8 GHz to 9.5 GHz) frequency bands. The prototypes of the proposed attenuator and phase shifter are fabricated using an LPKF micromilling machine on a Rogers RO4003C substrate with $\:{\epsilon\:}_{r}$ =3.55, tan$\:\delta\:$=0.0027, thickness h = 0.813 mm, and metal thickness t = 18 μm. The microstrip transmission line width Wf = 1.94 mm is optimized to achieve 50Ω impedance. A tapered transition is adopted to obtain an optimal matching condition between the microstrip and half mode SIW transmission line. The half mode SIW transmission line has a slit in the center with a size of L×W = 8 mm×0.5 mm. The one-sided vias of a half mode SIW are filled with Voltera conductor 3 silver ink. To increase the conductivity of the ink, the vias need are dried on a hot plate at 50 °C for 10 min. The graphene pad is positioned in the center and at the open side of the half mode SIW line, where the strength of the electric field distribution is maximum. The small gap of 1 mm×0.3 mm is adjusted according to the aspect ratio^13^ and^23^ for the deposition of graphene. Graphene nanoplatelets used in this work are from Tokyo Chemical Industries (TCI), with model number G0438, and have a 6–8 nm thickness and an average width of 25 μm.

**Fig. 8:**
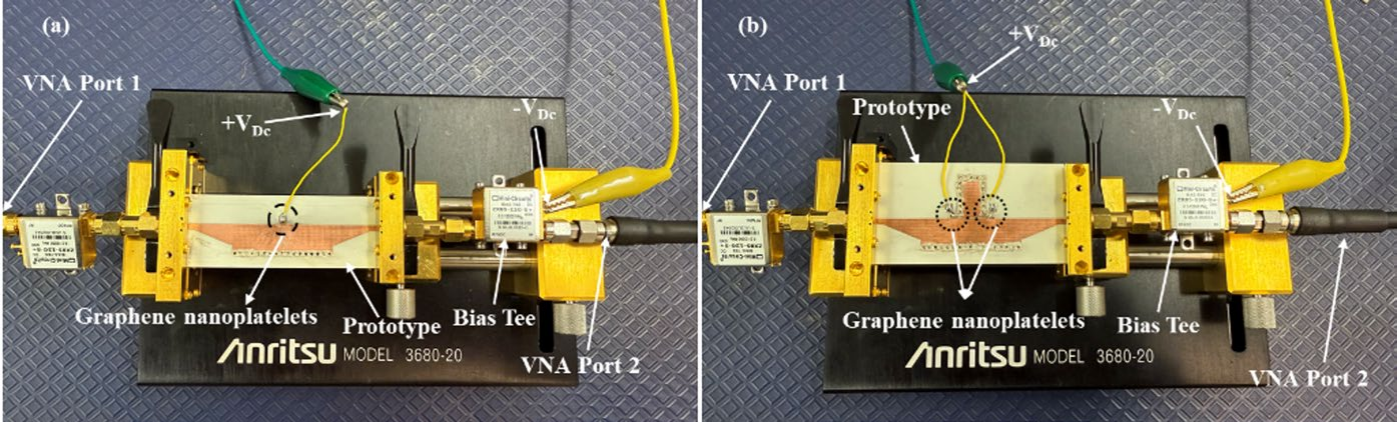
Fabricated prototype and measurement setup (**a**) GHMSIW attenuator (**b**) GHMSIW Phase shifter.

**Fig. 9:**
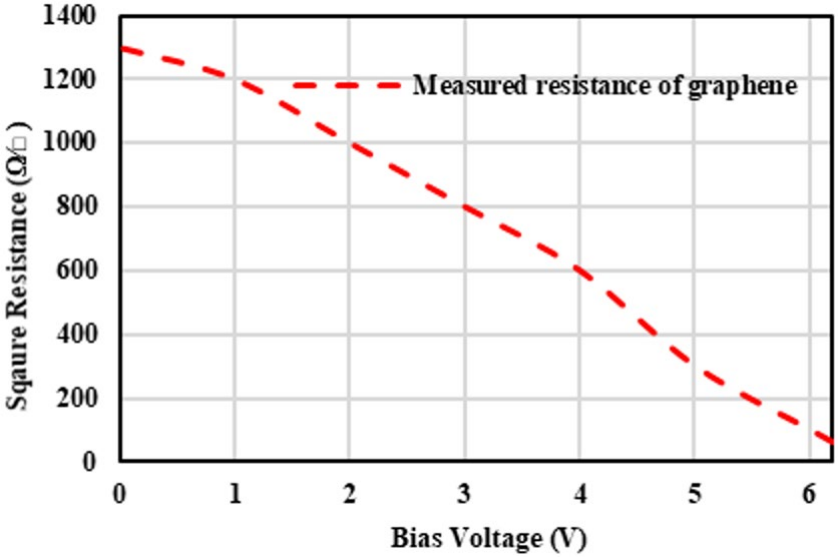
Graphene resistance versus applied voltage.

The measurement setup of the GHMISW attenuator and phase shifter comprises a 3680-test fixture from Anritsu, a bias tee from minicircuits ZX85-12G connected to port 1 and port 2 of a VNA (R&S ZVA24-1145111026) as shown in Fig. 8 (a) and (b). The prototype of the GHMSIW attenuator and phase shifter has been tested, and the measurement setup was calibrated using Thru Open Short Matched (TOSM) calibration standards. The biasing voltage is supplied to the deposited graphene by connecting a DC voltage between the biasing pad and the half mode SIW transmission line. The biasing pad near the gap is used for providing a positive voltage while the negative voltage is provided by the bias tee connected to the microstrip line. The main half-mode SIW transmission line is separated from the full-mode SIW stub with a gap of 0.3 mm. The full mode SIW stub is positioned in the center and at the open side of the half mode SIW line. The two graphene pads are biased separately with biasing lines connected to biasing pads. The two graphene pads are in parallel to each other, resulting in a combined reduced resistance value. For each value of DC voltage Vdc, the values of scattering parameters in the 5.5–9.5 GHz frequency band are measured. The applied voltage is gradually increased (0 V to 6.2 V). The resistance of graphene decreases (1300 Ω/□ to 60 Ω/□) with an increase in the DC bias voltage, hence reducing the attenuation and altering the phase of the signal between port 1 and port 2 as shown in Fig. 9.

## Results and discussions of the attenuator and phase shifter

**Fig. 10:**
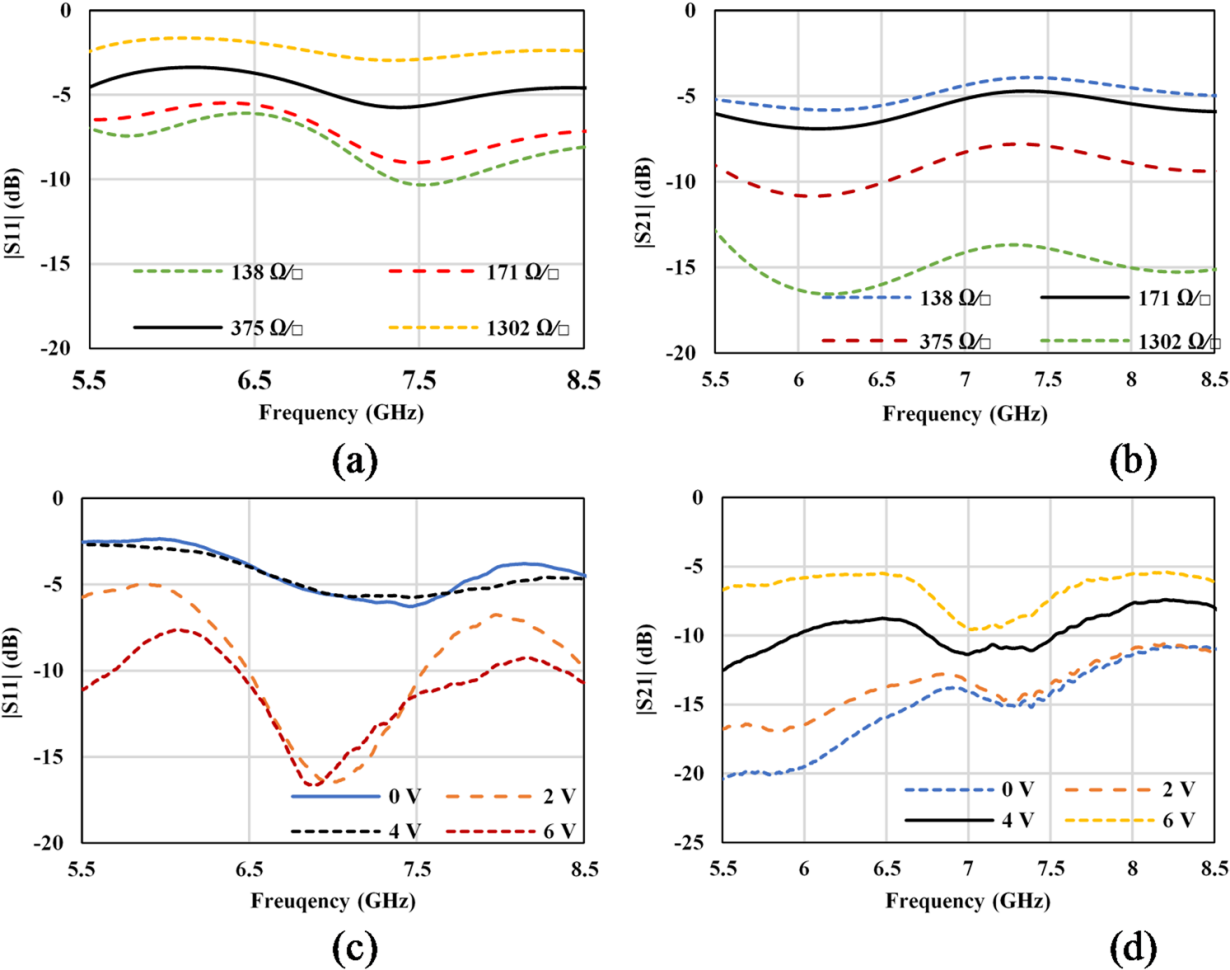
Simulated and measured results of the proposed attenuator (**a**) simulated amplitude of S_11_ (**b**) measured amplitude of S_11_, (**c**) simulated amplitude of S_21_ (**d**) measured amplitude of S_21_.

The design of the GHMSIW attenuator is optimized with the help of simulations to make a half mode SIW transmission line as a first attempt. Then the gap is introduced and optimized to achieve the maximum tuning range of the transmission coefficient (S_21_) of the attenuator, as shown in Fig. 10 (b). It is clear from Fig. 10 (a) that with an increase in voltage, the resistance of graphene decreases; hence, the reflection coefficient goes below − 10 dB (138 Ω/□), enhancing the transmission of the signal between the two ports of the attenuator. The graphene sheets were assigned as impedance boundaries with the real part Rg in the simulations. The simulations are performed for different values of graphene impedance, ranging from 138 Ω/□ to 1302 Ω/□. During the measurement of the attenuator, the DC resistance of graphene was measured as a function of the applied bias voltage. The simulated and measured values of the reflection coefficient are shown in Fig. 10 (a) and (c), respectively. The reflection coefficient is lower than − 5dB in simulations and measurements. With the increase in voltage, impedance is decreased, which causes an increased transmission of the signal from port 1 to port 2 of the attenuator. The measured insertion loss varies from 20 dB (when Vbias = 0) to 5.45 dB (when Vbias = 6 V), with a total dynamic range of 13 dB. The attenuation (dynamic range) in the transmitted signal is higher in the lower frequency band (5.5–7 GHz), and the attenuation (dynamic range) decreases with an increase in frequency (7–8.5 GHz). This behavior is characteristic of multilayered graphene, also present in^25^. The maximum insertion loss is 5.45dB at the center frequency of 8 GHz, as depicted in Fig. 10 (d). The discrepancy in the simulated and measured results of the attenuator can be attributed to the modelling of graphene in the FEM tool and in part due to fabrication errors.

Table 1. shows a comparison of the proposed GHMSIW attenuator with the state of the art of attenuators. This is a first attempt in SIW-based technology exploiting the tunable behavior of multi-layered graphene in attenuator applications; therefore, a comprehensive table based on different technologies is compiled.

**Table 1:** Comparison of the proposed Ghmsiw attenuator with the previously reported tunable attenuators.

Ref.	Size mm^2^	Freq Band (GHz)	ΔIL (dB)	Min IL (dB)	Technology
^7^	0.91λ × 0.45λ	0–5	14	0.3	Microstrip+ FLG*
^16^	2.5λ × 0.83λ	7-14.5	13	2.5	SIW + Monolayer graphene (GSS)
^26^	5.14λ × 1.28λ	7.7–19	12	3	HMSIW + Monolayer graphene (GSS*)
^27^	3.04λ × 1.40λ	1–7	6	1	HMSIW*+π N/W* resistor
^28^	1.33λ × 0.53λ	6–10	6	2.5	HMSIW + PIN diode
This work	1.51λ × 0.58λ	5.5–8.5	13	5.45	HMSIW + FLG

N/W*=Network, FLG*=Few layers graphene, GSS*=Graphene sandwiched structure, HSMIW = Half mode substrate integrated waveguide.

The improvement in terms of higher attenuation with less reflection is desirable. In future design, we will apply a strategy to achieve higher attenuation, lower phase error, and lower reflection coefficient in the targeted frequency band.

**Fig. 11:**
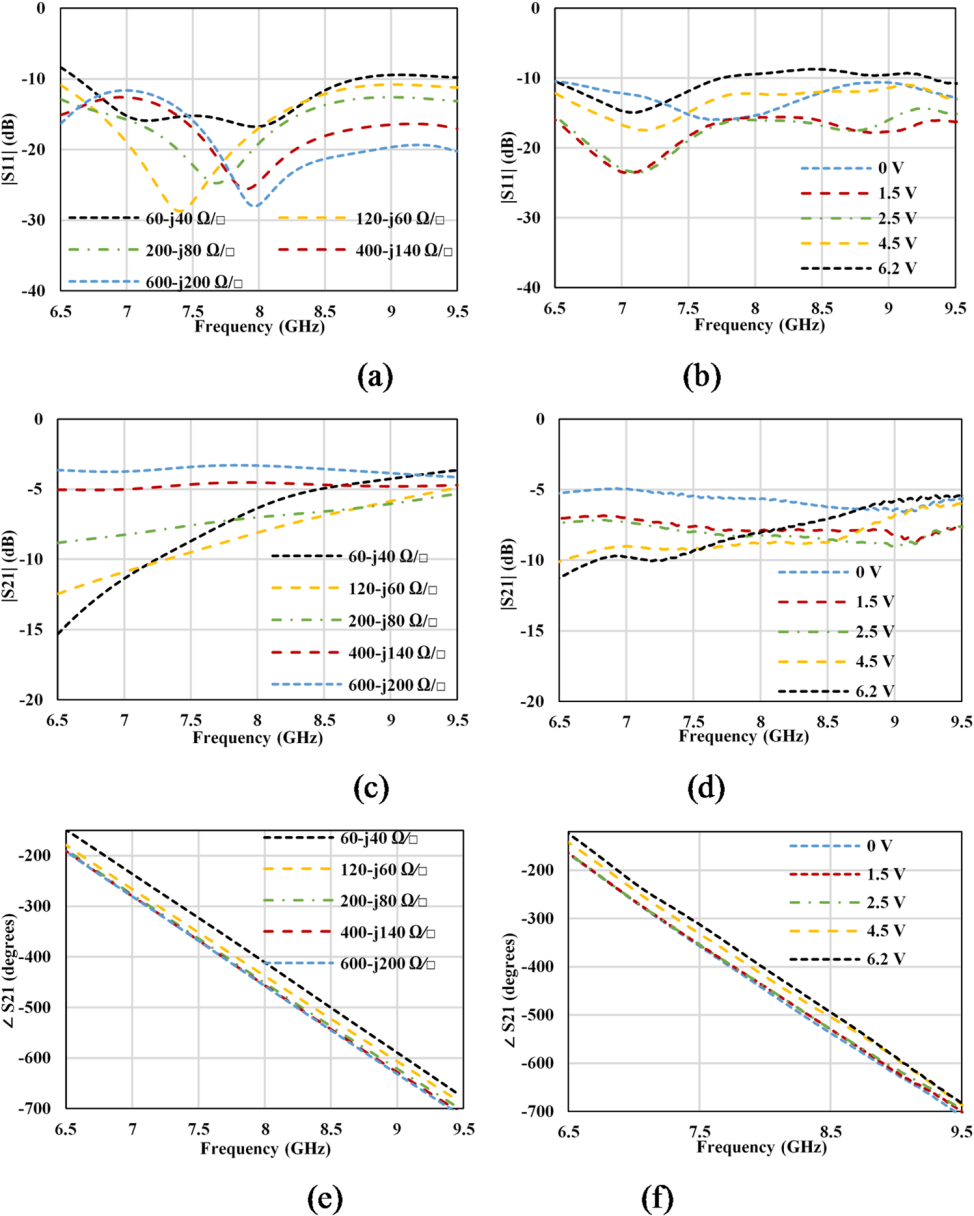
Simulated and measured results of the proposed PS (**a**) simulated amplitude of S_11_ (**b**) measured amplitude of S_11_, (**c**) simulated amplitude of S_21_ (**d**) measured amplitude of S_21_, (**e**) simulated phase of S_21_, (**f**) measured phase of S_21_.

The widths and lengths of the different sections of the GHMSIW are optimized with the help of simulations to maximize the phase variation and minimize the amplitude variation of the transmission coefficient. The graphene sheets were assigned as impedance boundaries with the real part Rg and the imaginary part Xg in the simulations. The simulations are performed for different values of graphene impedance, ranging from 600-j200 Ω∕□ to 60-j40 Ω∕□. DC sheet resistance in 600, 400, 200, 120, and 60 Ω∕□, used in simulation, corresponds to the voltage measured in volts 6.2, 4.5, 2.5, 1.5, and 0 V, respectively. It is worthwhile to note here that the values of reactance are considered here for the first time in the simulations of a phase shifter. During the measurement of the phase shifter, the DC resistance of graphene versus the applied bias voltage was measured. The simulated and measured values of the reflection coefficient are shown in Fig. 11 (a) and (b), respectively.

The reflection coefficient is lower than − 10dB in simulations as well as in measurements. With the increase in voltage, impedance is decreased, which causes an increased delay of the signal from the main half mode SIW line to the full-mode SIW stub. The increased delay causes an increased phase variation but also augments both the transmission and reflection losses. The maximum measured insertion loss varies from 4.9 dB (when Vbias = 0) to 12 dB (when Vbias = 6.2 V) whereas the phase shift is 45 degrees over the entire frequency band as depicted in Fig. 11 (c), (d), (e), and (f). The measured variation in the phase of the transmission coefficient versus the variation in amplitude amounts to almost 20°/dB: (∆∠S21)/(∆|S21|) = 20°/dB. The discrepancy in the measured values in comparison with the simulated values can be attributed to the inconsistencies in material deposition, PCB fabrication tolerances and manufacturing errors.

**Table 2:** Comparison of the proposed phase with the other technologies phase shifters.

Ref.	Size mm^2^	Freq Band (GHz)	Δ|S_21_| (dB)	Phase (deg)	FOM* (deg/dB)		Technology
^4^	-	4-4.5	2	23		54.7	IDC + Graphene
^17^	1.33λ × 0.6λ	4.4–6.2	1.5	60		-	SIW + PIN diode
^19^	5.2λ × 3.3λ	6–12	2.3	180		-	SIW + LM
^29^	1.17λ × 0.22λ	9.7–10.2	3.3	97		29	BST* thin film
^30^	3λ × 1λ	8–14	1.2	41–180		163	HMSIW + LM
^31^	-	8–12	1.53	60		12	Microstrip + Graphene
^32^	-	4–6	1	59		-	Microstrip + Graphene
This work	1.73λ × 0.67λ	6.5–9.5	2.25	45		20	HMSIW + Graphene

*BST = barium strontiusm titanate, *FOM = figure of merit.

Table 2 compares the proposed GHMSIW phase shifter with the state of the art of phase shifters. This is a proof of concept and a first attempt in SIW-based technology exploiting the tunable behavior of multi-layered graphene in phase shifting applications; therefore, a comprehensive table based on similar technology could not be compiled. The performance of the proposed phase shifter in terms of the insertion loss and phase variation is comparable to some in the state of the art. Additionally the proposed phase shifter is advantageous in terms of ease of integration, broadband, ease of fabrication, and compactness compared to conventional SIW phase shifters.

**Fig. 12:**
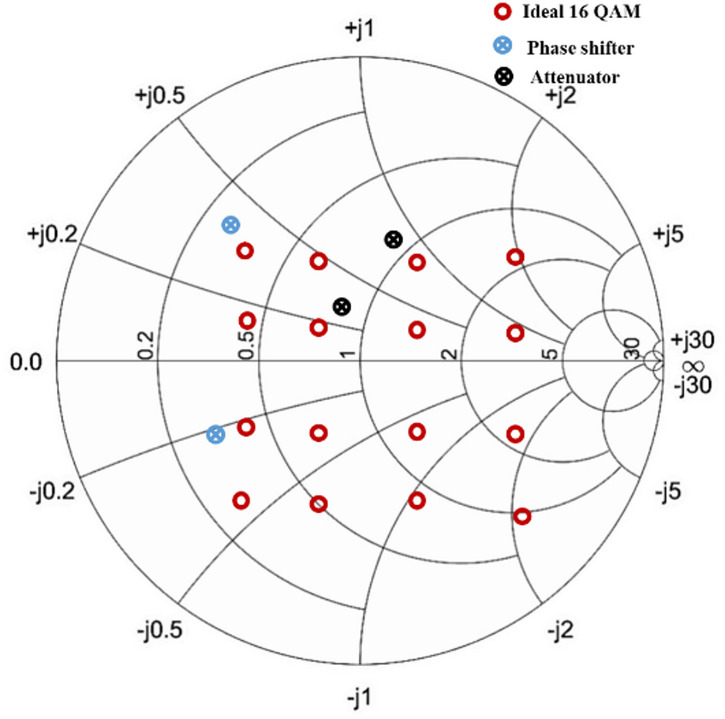
Ideal 16 QAM on smith chart at 8 GHz with the possible amplitude and phase variation at 100 and 1300 Ω∕□.

The amplitude and phase variations achieved by the SIW attenuator and phase shifter can be plotted on a smith chart, represented by black and blue circles respectively as shown in Fig. 12. The points of amplitude and phase are taken at a center frequency of 8 GHz and represent amplitude and phase for resistance of graphene of 100 Ω∕□ and 1300 Ω∕□. The relevance of the amplitude and phase variation is highlighted by the fact that it can achieve considerable values close to those of a reflection type 16 QAM represented by red circles. These values have been derived from circuit simulations of a reflection type power divider based QAM. This demonstrates that an enhanced version of the proof of concept SIW attenuator and phase shifter can be extended to develop an SIW IQ modulator. The concept explained in this work will bring a paradigm shift in implementing multilayered graphene in substrate integrated waveguide-based microwave attenuators and phase shifters.

## Conclusion

A novel multilayered graphene based half-mode substrate integrated waveguide (GHMSIW) microwave components for amplitude and phase manipulation of electromagnetic waves are presented. In contrast to conventional electronic elements like PIN or varactor diodes, multilayered graphene is chosen as a tunable element in the SIW based attenuator and phase shifter. The multilayered graphene is deposited in such a way that maximum manipulation (amplitude and phase) of electromagnetic waves is achieved. Multilayered graphene surface conductivity is tuned by applying a voltage from 0 to 6.2 V. The attenuator and phase shifter are capable of attaining the amplitude attenuation in total up to -13dB and phase shift up to 45 degrees of the transmitted signal between ports 1 and 2. The design of the attenuator and phase shifter is simple, compact, low cost, and easy to integrate with 2D structures for microwave applications. The integration of these two components in a single structure as an amplitude and phase control module will make the system more compact. The work proposed in this paper advances the state of the art for multilayered graphene-based SIW devices or systems.

## Data Availability

The datasets used and/or analyzed during the current study are available from the corresponding author on reasonable request.
